# Global Prevalence and Distribution of Human Sarcocystosis: A Systematic Review and Meta-Analysis

**DOI:** 10.3390/tropicalmed11090241

**Published:** 2026-08-25

**Authors:** Jurairat Jongthawin, Aongart Mahittikorn, Kinley Wangdi, Frederick Ramirez Masangkay, Manas Kotepui

**Affiliations:** 1 Faculty of Medicine, Mahasarakham University, Mueang Maha Sarakham 44000, Thailand; jurairat.j@msu.ac.th; 2 Department of Protozoology, Faculty of Tropical Medicine, Mahidol University, Bangkok 10400, Thailand; 3 HEAL Global Research Center, Health Research Institute, Faculty of Health, University of Canberra, Canberra, ACT 2617, Australia; kinley.wangdi@canberra.edu.au; 4 National Centre for Epidemiology and Population Health, College of Law, Governance and Policy, Australian National University, Canberra, ACT 2601, Australia; 5 Department of Medical Technology, Faculty of Pharmacy, University of Santo Tomas, Manila 1008, Philippines; frmasangkay@ust.edu.ph; 6 Research Center for the Natural and Applied Sciences, University of Santo Tomas, Manila 1008, Philippines; 7 Medical Technology Program, Faculty of Science, Nakhon Phanom University, Mueang Nakhon Phanom 48000, Thailand

**Keywords:** *Sarcocystis*, protozoa, intestinal parasites, systematic review, meta-analysis, global distribution, sarcocystosis

## Abstract

Human sarcocystosis is a neglected zoonotic infection caused by protozoa of the genus *Sarcocystis*. Although sporadic cases and outbreaks have been reported worldwide, the global burden of human infections has not been comprehensively synthesized. This study aimed to estimate the pooled prevalence and global distribution of *Sarcocystis* infections in humans. A systematic review and meta-analysis were conducted in accordance with PRISMA guidelines and registered with PROSPERO (CRD420251159944). PubMed, Scopus, Web of Science, Ovid, Nursing & Allied Health Premium, and Google Scholar were used for retrieving relevant studies. Observational studies published from 2000 onward reporting human *Sarcocystis* infections confirmed by microscopic and/or molecular methods were included. A random-effects model was used to estimate pooled prevalence. Heterogeneity was assessed using the *I*^2^ statistic. Subgroup analyses and meta-regression were performed to explore sources of heterogeneity. Seventeen studies comprising 66,329 participants met the inclusion criteria. The pooled prevalence of human *Sarcocystis* infection was 0.85% (95% confidence interval [CI]: 0.33–2.19), with substantial heterogeneity (*I*^2^ = 98.2%). Prevalence estimates showed marked variation across continents, countries, participant groups, diagnostic methods, *Sarcocystis* species, and clinical forms of sarcocystosis. Although human *Sarcocystis* infection appears relatively rare, its prevalence is geographically heterogeneous across studies published between 2000 and 2024. The robustness of the pooled prevalence estimate is limited by the small number of studies, uneven geographical coverage, and substantial heterogeneity. Differences in diagnostic approaches, study populations, infecting species, and disease phenotypes likely contributed to this variability. Enhanced surveillance and standardized application of sensitive molecular diagnostics are essential to better define the epidemiology, burden, and public health significance of human sarcocystosis.

## 1. Introduction

Sarcocystosis is a widespread but neglected zoonotic infection caused by coccidian protozoa of the genus *Sarcocystis* (Phylum Apicomplexa). These obligatory intracellular parasites maintain a complex two-host life cycle involving an intermediate host (prey) and a definitive host (predator) [1,2]. Humans occupy a unique epidemiological niche in this cycle, capable of serving either as definitive hosts for specific species or as accidental intermediate hosts for others, resulting in two distinct clinical syndromes: intestinal and muscular sarcocystosis [3].

Human intestinal sarcocystosis is the more common presentation, occurring when humans serve as definitive hosts after ingesting raw or undercooked meat containing mature sarcocysts. This form is primarily attributed to *Sarcocystis hominis* (acquired from beef) and *Sarcocystis suihominis* (acquired from pork) [4]. While often asymptomatic or presenting with mild, self-limiting gastroenteritis (nausea, abdominal pain, and diarrhea), the infection can persist, leading to chronic shedding of infective sporocysts into the environment [5,6].

In contrast, human muscular sarcocystosis represents a more severe, albeit historically sporadic, form of disease. Here, humans serve as accidental intermediate hosts after ingesting water or food contaminated with sporocysts or oocysts shed by definitive hosts (likely reptiles or wild carnivores) [3,7]. This infection results in the formation of sarcocysts within skeletal and cardiac muscles, characterized by acute fever, myalgia, vasculitis, and localized muscle swelling [8]. The emergence of *Sarcocystis nesbitti* as a causative agent has recently garnered significant attention following large-scale outbreaks among tourists and residents in Southeast Asia, particularly in Malaysia, underscoring the pathogen’s evolving public health relevance [7,9].

Despite its global distribution, human sarcocystosis remains significantly underreported and diagnostically challenging. The disease has led to significant economic losses in the livestock industry, including production losses resulting from abortion, reduced meat quality, and carcass damage in intermediate hosts such as sheep, goats, and cattle [10]. Furthermore, human sarcocystosis poses a public health risk because humans can serve as definitive hosts for several *Sarcocystis* species; among these, *S. hominis* and *S. suihominis* are considered the main species of public health importance and are acquired through the consumption of undercooked beef and pork, respectively [1,3]. The routine diagnostic method, stool microscopy, is hindered by low sensitivity and cannot morphologically differentiate between *Sarcocystis* species [4,11,12]. Furthermore, muscular sarcocystosis is often misidentified as other tropical febrile illnesses because definitive diagnosis typically requires invasive muscle biopsy or advanced molecular confirmation, which are not routinely available in resource-limited settings [13,14]. While recent systematic reviews have mapped the prevalence of *Sarcocystis* spp. in livestock reservoirs such as cattle [15] and small ruminants [16], a comprehensive synthesis of the global burden in humans is notably absent. Current knowledge relies largely on fragmented case reports, localized outbreaks, and outdated prevalence surveys, making it difficult to estimate the true scope of human infection. To address this gap, this study provides a systematic review and meta-analysis of *Sarcocystis* infection in humans. By synthesizing data from observational studies worldwide, this study aim to estimate the global pooled prevalence of human sarcocystosis, analyze geographical and demographic variations, and critically evaluate the influence of diagnostic methodologies on reported infection rates.

## 2. Methods

### 2.1. Registration and Protocol

The systematic review and meta-analysis were registered with PROSPERO (ID: CRD420251159944) and reported according to the Preferred Reporting Items for Systematic Reviews and Meta-Analyses (PRISMA) guidelines [17].

### 2.2. Definitions

*Sarcocystis* infections in this study were defined as infections caused by any *Sarcocystis* species infecting humans and were detected in fecal or biopsy samples using microscopic or molecular methods.

### 2.3. Eligibility Criteria

Studies were considered eligible for inclusion if they reported human *Sarcocystis* infections and provided original data for synthesis. Eligible studies included observational designs such as cross-sectional or cohort studies that investigated *Sarcocystis* infection in human populations. Studies were required to confirm or detect human *Sarcocystis* infection using recognized diagnostic methods, including microscopic, molecular, histological, immunological, and/or serological methods, using appropriate clinical specimens such as fecal, biopsy, blood, or other relevant human specimens. The eligibility criteria were also restricted to studies published from 2000 onward to focus on contemporary evidence and diagnostic approaches relevant to human *Sarcocystis* infection. Studies were excluded if they were review articles, case reports, case series, in vitro studies, experimental infections, comments, or test performance evaluations. Articles focusing on *Sarcocystis* in animals, food products, or environmental samples were also excluded, as were conference abstracts and book chapters.

### 2.4. Search Strategy

A comprehensive and systematic literature search was conducted to identify studies reporting human *Sarcocystis* infections. The search strategy was developed using a combination of controlled vocabulary terms (when available) and free-text keywords to maximize sensitivity and ensure broad coverage of relevant literature. The primary search terms focused on the parasite and the human host and included the keywords “*Sarcocystis*,” “Sarcocysti,” “Sarcosporidia,” and “Sarcosporidias” combined with “human” or “patients” using Boolean operators. Electronic searches were performed in PubMed, Scopus, Web of Science, Ovid (Journal), and Nursing & Allied Health Premium, with all databases searched up to 30 September 2025. In PubMed, both Medical Subject Headings (MeSH) and keywords were applied to capture indexed and non-indexed records. The detailed search terms, database-specific strategies, and the number of records retrieved from each source are provided in Table S1. An additional Google Scholar search was conducted to identify potentially relevant studies that may have been missed during the primary literature search.

### 2.5. Study Selection and Data Extraction

All retrieved records were exported and managed collectively using EndNote version 21.0 (Clarivate Analytics, Philadelphia, PA, USA), and duplicate records were removed prior to screening. After removing duplicate records, all remaining records were screened by title and abstract to assess relevance to human *Sarcocystis* infection. Records that did not meet the predefined scope were excluded at this stage. Full-text articles were then retrieved for potentially relevant records and assessed against the eligibility criteria. Studies that did not meet the inclusion requirements were excluded following full-text evaluation. The studies that satisfied all eligibility criteria were finally included in the systematic review. The study selection was conducted independently by two authors (MK, AM), and any disagreements were resolved through discussion to reach consensus.

For each included study, key information was extracted and summarized in Microsoft Excel 2021 (Microsoft Corporation, Redmond, WA, USA). Extracted data included bibliographic details (first author), methodological characteristics (study design, year of study, and study location), and geographical information (continent). Participant-related data were also collected, including participant type, age range, proportion of male participants, total sample size, and the number of confirmed *Sarcocystis* infections. In addition, diagnostic variables, including the method used for *Sarcocystis* detection, the type of specimen analyzed, the identified *Sarcocystis* species, and the target gene used for molecular detection, were extracted when applicable. Data extraction was performed systematically for all studies that met the inclusion criteria by one author (MK) and cross-checked by another author (JJ).

### 2.6. Risk of Bias

The methodological quality of the included studies was assessed using the Joanna Briggs Institute (JBI) Critical Appraisal Checklist for Prevalence Studies [18]. This tool evaluates nine domains, including sample frame appropriateness, sampling method, sample size adequacy, description of the study setting, data analysis coverage, validity and reliability of outcome measurement, statistical analysis, and response rate. Each item was rated as “Yes,” “No,” or “Unclear.” Studies were categorized as low, moderate, or high risk of bias based on the proportion of criteria fulfilled, as suggested by a previous study [19]. Two authors (MK and JJ) independently assessed the risk of bias, and discrepancies were resolved through consensus.

### 2.7. Data Syntheses

The pooled prevalence of *Sarcocystis* infections in humans was estimated using a random-effects model to account for heterogeneity among the included studies [20]. Heterogeneity was assessed using the *I*^2^ statistic, with values exceeding 50% indicating substantial heterogeneity [21]. Subgroup analyses and meta-regression were performed to explore potential sources of heterogeneity. Publication bias was evaluated through visual inspection of funnel plot asymmetry and formally assessed using Egger’s test when at least 10 studies were included in the meta-analysis. Sensitivity analysis using a fixed-effect model was conducted to assess the robustness of the pooled estimates. All analyses were performed using RStudio (version 2023.09.1 + 494) [22].

## 3. Results

### 3.1. Search Results

**Figure 1:**
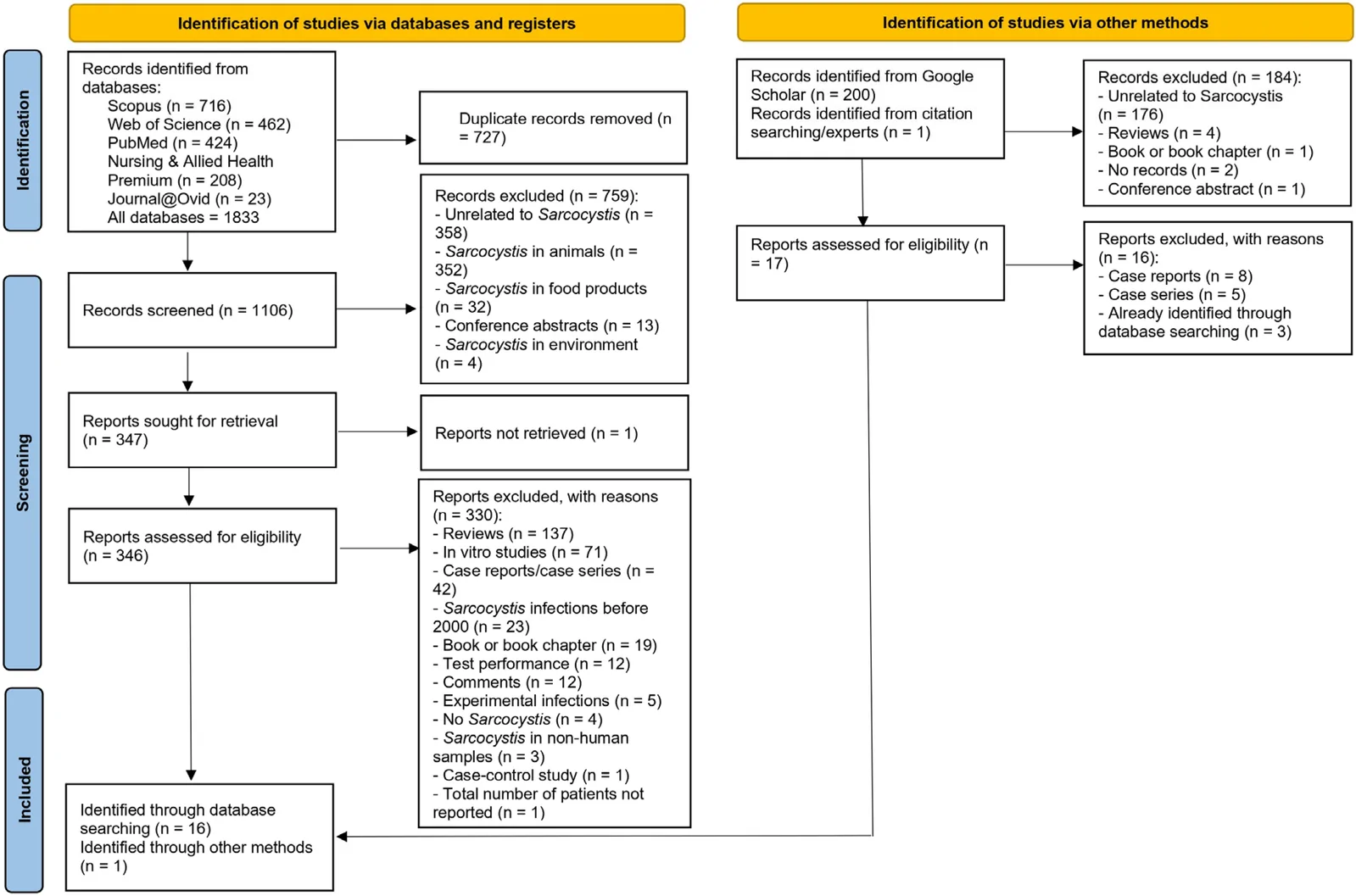
The flow diagram demonstrates the selection of studies for inclusion in the systematic review.

A total of 1833 records were identified through searches of five major databases, with an additional 200 records identified through Google Scholar and one record identified through citation searching or consultation with experts in the field. After removing 727 duplicate records, 1106 records remained for title and abstract screening. During this stage, 759 records were excluded because they were unrelated to *Sarcocystis*, including animal records, food products, environmental samples, or conference abstracts. The remaining 347 reports were sought for retrieval; one could not be retrieved. A full-text eligibility assessment was therefore conducted on 346 reports, resulting in the exclusion of 330 reports for reasons including review articles, in vitro studies, case reports or case series, publications prior to 2000, book chapters, test-performance studies, comments, experimental infections, studies without *Sarcocystis*, studies involving non-human samples, case-control studies, and the total number of patients not reported. Through the database-searching pathway, 16 studies met the inclusion criteria. In addition, 17 reports identified through Google Scholar, citation searches, and expert recommendations were assessed for eligibility; 16 were excluded, including 3 already identified through database searches, resulting in 1 additional study. Thus, a total of 17 studies [4,5,7,8,11,12,13,14,23,24,25,26,27,28,29,30,31] were included in the systematic review (16 identified through database searching and 1 through other methods) (Figure 1).

### 3.2. Characteristics of Included Studies

A total of 17 studies were included in the systematic review [4,5,7,8,11,12,13,14,23,24,25,26,27,28,29,30,31]. Most studies employed a cross-sectional design (n = 16, 94.4%) [4,5,7,8,11,12,13,14,23,24,25,26,27,28,29,30,31]. The studies were conducted between 2007 and 2024, and all were published after 2010. Geographically, most studies were conducted in Asia (13 studies, 76.5%) [5,7,8,12,13,14,23,24,25,26,27,28,30], particularly in Malaysia [7,8,13,14,27,28], Iran [23,25,30], Thailand [5,12,24], and Cambodia [26]. Additional studies were conducted in Europe, including Belgium [4], France [29], Luxembourg [29], Italy [31], as well as in North America (Mexico) [11]. The majority of studies enrolled villagers [5,24,26,27,28], and hospital/clinics-based patients [4,8,11,13,29,31], symptomatic college students and teachers [7,14], schoolchildren [30], and immunocompromised individuals [23], spanning a wide age range from early childhood to older adulthood. Fecal samples were the most analyzed specimens [4,5,11,12,13,23,24,25,26,27,28,29,30,31], while the remaining studies used biopsy samples [7,8,14]. The majority of included studies employed direct microscopy-based methods for the detection of *Sarcocystis* infections [4,5,11,12,24,25,26,27,28,30], whereas the remaining studies used microscopy-based methods combined with polymerase chain reaction (PCR) [23,29], PCR alone [7,14], Semi-nested PCR combined with sequencing [31], histology combined with PCR [8], or an immunofluorescent antibody test [13] (Table S2).

### 3.3. Risk of Bias Results

The methodological quality of the included studies was evaluated using the JBI Critical Appraisal Checklist for Prevalence Studies. Overall, three studies [7,26,29] were classified as low risk of bias, ten [5,11,12,13,14,23,24,25,27,30] as moderate risk, and four [4,8,28,31] as high risk. Most studies demonstrated appropriate diagnostic methods, adequate description of study settings, and suitable statistical analyses. However, limitations were commonly observed in the sampling methodology, justification of sample size, and reporting of response rates (Table S3).

### 3.4. Prevalence of Sarcocystis Infections in Humans

**Figure 2:**
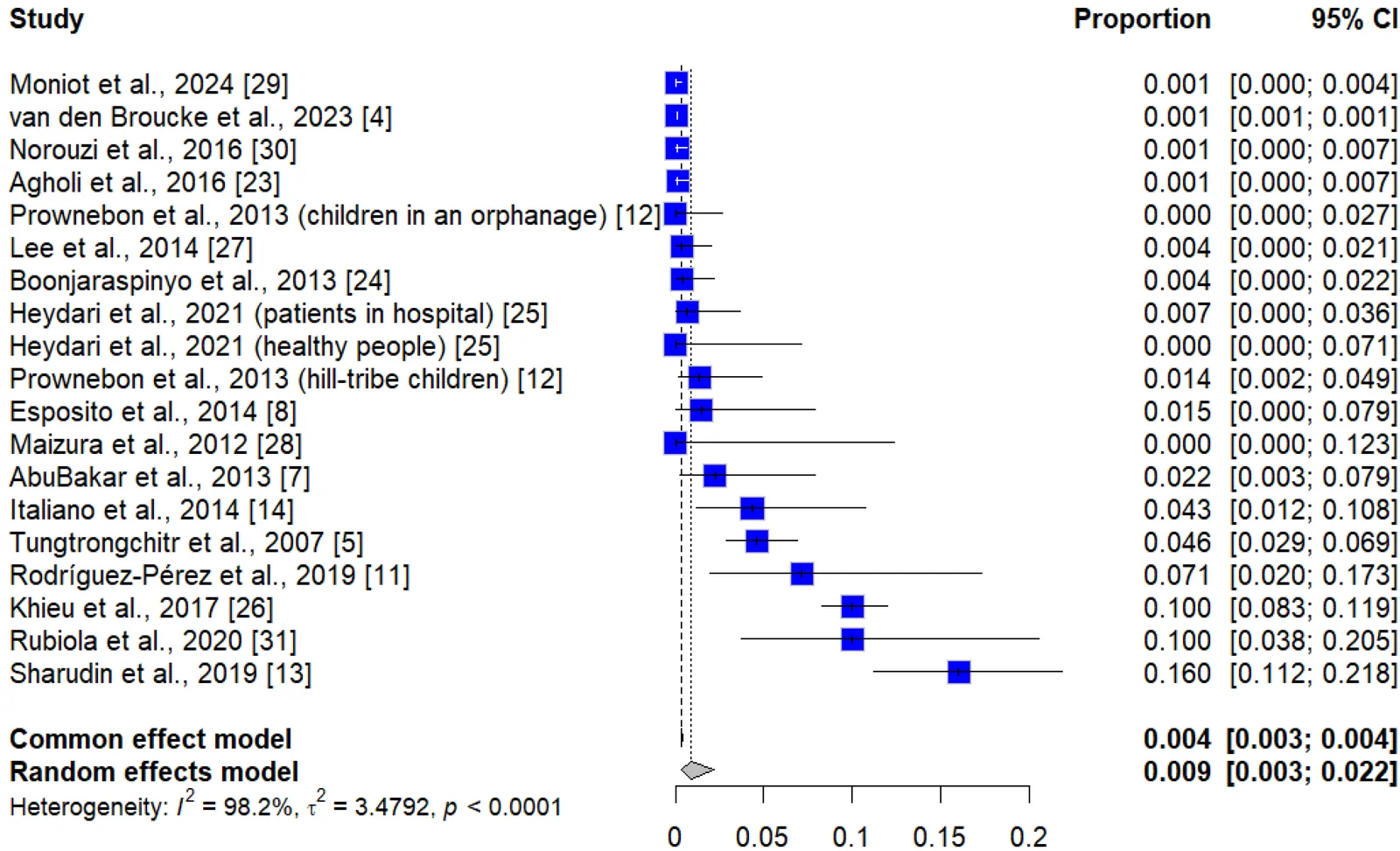
Forest plot of the prevalence of *Sarcocystis* infection in humans across included studies. Each square represents the prevalence estimate reported by an individual study. Horizontal lines indicate the 95% confidence intervals (CIs). The diamond symbols represent the pooled prevalence estimates derived from the common-effect and random-effects models [4,5,7,8,11,12,13,14,23,24,25,26,27,28,29,30,31].

The prevalence of *Sarcocystis* infections in humans was 0.85% (95% CI: 0.33–2.19, *I*^2^: 98.2%, 17 studies with 66,329 participants, Figure 2) as estimated by the random-effects model.

The meta-regression analysis using continent, country, participants, age group, method for *Sarcocystis* detection, *Sarcocystis* species, and types of *Sarcocystis* infection showed that country and method for *Sarcocystis* detection significantly affected the pooled prevalence of *Sarcocystis* infections in humans (*p* < 0.05, Table S4).

Subgroup analysis by geographic region showed significant differences in the pooled prevalence of *Sarcocystis* infections (*p* = 0.0067). The pooled prevalence in Asia was 0.94% (95% CI: 0.34–2.55%) across 15 datasets, with substantial heterogeneity (*I*^2^ = 86.8%). In North America, the pooled prevalence was 7.14% (95% CI: 2.71–17.54%; 1 dataset), whereas in Europe it was 0.38% (95% CI: 0.02–5.52%; 3 datasets).

Country-level subgroup analysis also demonstrated significant differences in pooled prevalence (*p* < 0.0001). The highest pooled prevalence was observed in Italy (10.0%, 95% CI: 4.56–20.53%), followed by Cambodia (9.99%, 95% CI: 8.34–11.93%) and Mexico (7.14%, 95% CI: 2.71–17.54%), respectively. The pooled prevalence was 2.12% (95% CI: 0.59–7.35%) in Malaysia, 0.96% (95% CI: 0.21–4.30%) in Thailand, 0.17% (95% CI: 0.05–0.52%) in Iran, 0.10% (95% CI: 0.08–0.13%) in Belgium, and 0.06% (95% CI: 0.01–0.45%) in France and Luxembourg.

The pooled prevalence also differed significantly according to participant groups (*p* < 0.0001). The highest prevalence was observed among symptomatic college students and teachers (3.31%, 95% CI: 1.50–7.18%; 2 datasets), followed by villagers (1.46%, 95% CI: 0.30–6.88%; 5 datasets), patients in hospitals/clinics (1.23%, 95% CI: 0.24–6.18%; 7 datasets), and immunodeficient individuals (0.13%, 95% CI: 0.02–0.95%; 1 dataset). The pooled prevalence was 0.12% (95% CI: 0.02–0.83%, 1 dataset) among schoolchildren and 0.71% (95% CI: 0.18–2.79%, 1 dataset) among preschool-aged children. A prevalence of less than 0.00% (95% CI: 0.00–1.00%, 1 dataset) was observed among healthy individuals.

There was a significant difference in the pooled prevalence across age groups (*p* = 0.0060). The prevalence was 0.71% (95% CI: 0.18–2.79%; 2 datasets) among children and 7.14% (95% CI: 2.71–17.5%; 1 dataset) among adults. Studies including all age ranges had a pooled prevalence of 0.53% (95% CI: 0.13–2.21%; 8 datasets). Studies that did not specify age had a pooled prevalence of 1.24% (95% CI: 0.28–5.22%; 8 datasets).

The pooled prevalence differed significantly according to the method used to detect *Sarcocystis* (*p* < 0.0001). The highest prevalence was reported using immunofluorescent antibody testing (16.0%, 95% CI: 11.5–21.8%; 1 dataset), followed by semi-nested PCR/sequencing (10.0%, 95% CI: 4.56–20.5%; 1 dataset), histology/PCR (1.47%, 95% CI: 0.21–9.71%; 1 dataset), PCR (3.31%, 95% CI: 1.50–7.18%; 2 datasets), microscopic methods (0.61%, 95% CI: 0.19–1.97%; 12 datasets), and combined microscopic/PCR methods (0.09%, 95% CI: 0.02–0.35%; 2 datasets).

Significant differences were observed according to *Sarcocystis* species (*p* < 0.0001). The pooled prevalence was 2.81% (95% CI: 1.35–5.78%; 3 datasets) for *S. nesbitti*, 0.47% (95% CI: 0.06–3.35%; 5 datasets) for *S. hominis*, 0.13% (95% CI: 0.02–0.95%; 1 dataset) for *S. cruzi*, and 1.62% (95% CI: 0.39–6.41%; 8 datasets) for *Sarcocystis* spp.

The pooled prevalence differed significantly by type of *Sarcocystis* infection (*p* < 0.0001). The prevalence was 0.54% (95% CI: 0.18–1.65%) for intestinal infections (15 datasets), compared with 2.81% (95% CI: 1.35–5.78%) for muscular infections (3 datasets). The estimate for blood samples, in which the type of infection was not specified, was substantially higher, at 16.0% (95% CI: 11.54–21.75%; 1 dataset).

### 3.5. Publication Bias

**Figure 3:**
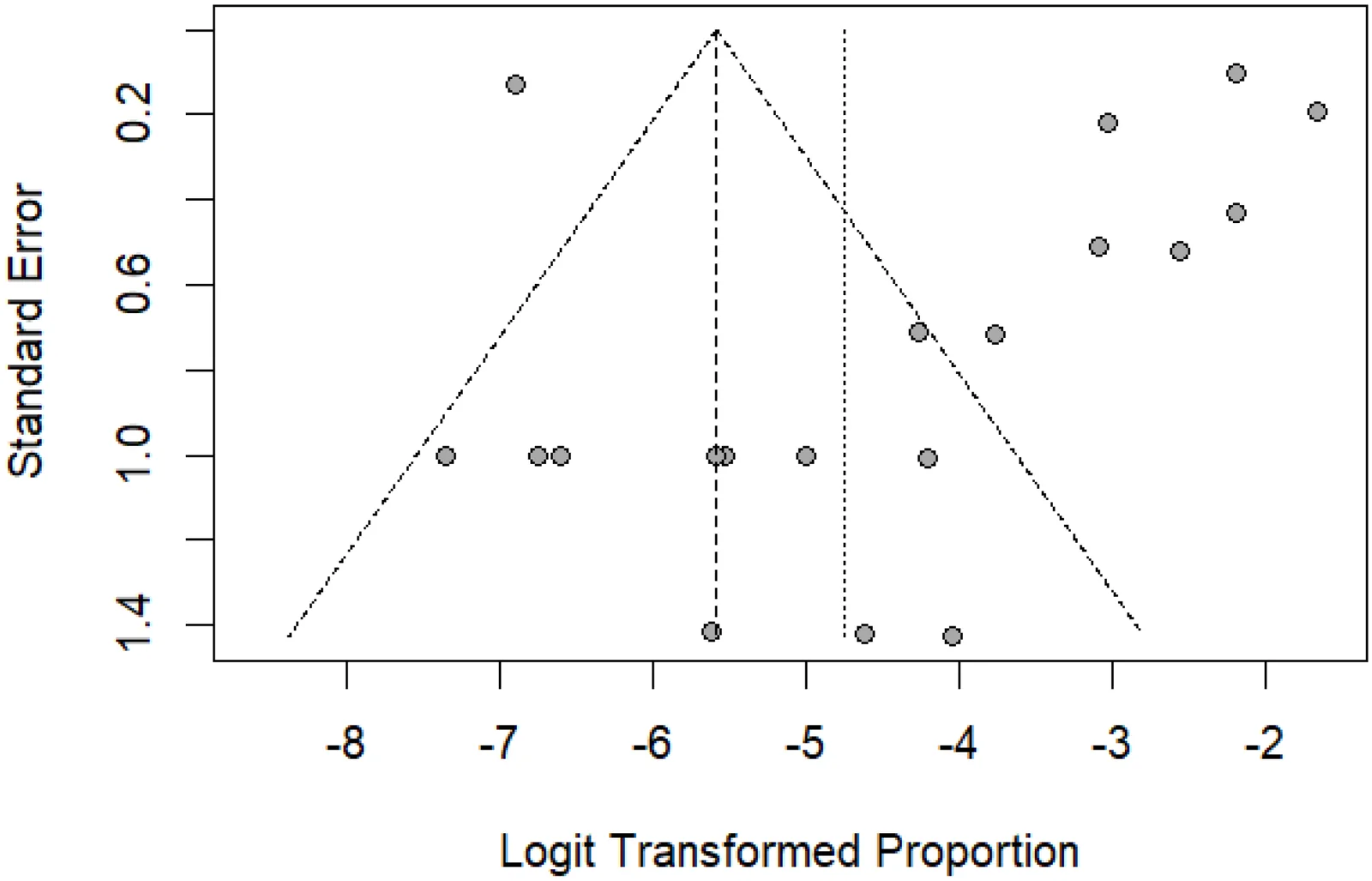
Funnel plot assessing publication bias in studies reporting the prevalence of *Sarcocystis* infection. Each point represents an individual study plotted by its standard error against the logit-transformed proportion. The dashed diagonal lines indicate the pseudo 95% confidence limits around the pooled estimate, and the vertical dashed line represents the overall pooled effect size.

The funnel plot assessing publication bias in studies reporting the prevalence of *Sarcocystis* infection showed visual asymmetry (Figure 3). However, Egger’s regression test did not detect statistically significant small-study effects (*p* = 0.6395, t = −0.48). This suggests that the observed funnel plot asymmetry is unlikely to be driven by publication bias and may instead reflect between-study heterogeneity or other methodological differences among the included studies.

## 4. Discussion

This systematic review and meta-analysis synthesized available evidence on the prevalence of human *Sarcocystis* infections and provides updated pooled estimates based on 17 studies encompassing over 66,000 participants. The overall pooled prevalence of 0.85% suggests that human sarcocystosis is relatively infrequent. However, the infection is geographically widespread and occurs in diverse populations. The very high heterogeneity observed across studies indicates substantial variability in study design, populations, and diagnostic approaches, which should be considered when interpreting the pooled estimates.

Most studies reporting *Sarcocystis* infections in humans were conducted in Asia, particularly in Malaysia, Iran, Thailand, and Cambodia. This geographical concentration may be partly explained by dietary habits, including the consumption of raw or undercooked beef and pork containing mature sarcocysts of *S. hominis* and *S. suihominis* [1]. The higher prevalence estimates reported in Cambodia and Mexico, compared with lower estimates in Iran and Thailand, suggest that country-specific factors strongly influence infection patterns. In Cambodia, reported cases were linked to wedding celebrations and Chinese New Year festivities, during which dishes containing raw or insufficiently cooked beef and pork were consumed [26]. In Mexico, infections were reported primarily among HIV-positive patients [11]. Similarly, studies from Iran documented *Sarcocystis* infections among immunocompromised individuals [23,25], suggesting that impaired immune responses may increase susceptibility to infection. Cell-mediated immunity, particularly CD4+ T-cell-mediated responses, plays a central role in controlling intracellular protozoan parasites [32]. This immune response led to the production of inflammatory mediators, including tumor necrosis factor (TNF), interferon-gamma (IFNγ), and interleukin-17 (IL-17), to control parasite growth [32]. In immunocompromised individuals, including those with HIV infection or receiving immunosuppressive therapy, reduced cellular immune function may impair parasite clearance, allowing prolonged survival or increased replication within host tissues. Nevertheless, *Sarcocystis* infections in Iran have also been reported among schoolchildren [30], indicating that dietary exposure, rather than immune status alone, may play an important role in transmission. Likewise, the low prevalence of *Sarcocystis* infection observed among Thai participants may be related to local eating practices in rural communities, which differ from those in higher-prevalence settings [24]. Geographical variation in the pooled prevalence should be interpreted with caution because only 17 studies were included, most of which were conducted in Asia. The higher prevalence observed in Asia may partly reflect the regional distribution of *S. nesbitti* and the availability and distribution of its definitive hosts, rather than differences in human exposure or infection risk alone.

Prevalence also varies by participant characteristics and age groups. Higher prevalence estimates among symptomatic college students, teachers, and adults compared with schoolchildren may reflect differences in exposure or health-seeking behavior. Although two studies reported a lower prevalence in children (0.71%) [12,30] compared with adults (7.14%) [11], *Sarcocystis* infection has been reported across a wide range of age groups [4,8,14,24,26,27,28]. This evidence suggests that human *Sarcocystis* infection is not clearly restricted to a particular age group and may be more strongly associated with dietary behaviors, particularly the consumption of undercooked beef or pork. A high prevalence in adults was observed in a study that enrolled patients at high risk of infection, such as hospitalized HIV-positive and HIV-negative immunocompromised patients with diarrhea [11]. Interestingly, the prevalence in villagers (1.46%) and in patients in hospitals/clinics (1.23%) was nearly identical. However, this similarity should be interpreted cautiously because the two populations differed in their clinical characteristics and reasons for study enrollment. The comparable prevalence estimates do not necessarily indicate that infections were asymptomatic in villagers and symptomatic in hospital or clinic patients. Rather, the findings may suggest that *Sarcocystis* infection can occur in both community and clinical populations.

Diagnostic methodology was a major source of variability among the included studies. Studies employing PCR-based methods (3.31%), semi-nested PCR combined with sequencing (10.0%), or immunofluorescent antibody testing (16%) reported higher prevalence estimates than those relying solely on microscopy-based techniques (0.61%). The higher prevalence observed in molecular-based studies may be attributed to the greater sensitivity of molecular methods for detecting low levels of *Sarcocystis* nucleic acids. In contrast, the elevated prevalence reported using immunofluorescent antibody testing may be explained by the use of biopsy specimens obtained from autopsy cases, including individuals who died from causes such as automobile accidents or cardiovascular diseases [13].

The pooled prevalence of intestinal infections (0.54%) was lower than that of muscular infections (2.81%), which may reflect differences in the detectability of *Sarcocystis* between fecal and muscle specimens, although the available data do not allow direct comparisons of diagnostic sensitivity. The limitations of conventional microscopy, particularly its lower sensitivity for detecting low parasite burdens, further highlight the potential value of molecular and serological methods for improving diagnostic accuracy in both stool and muscle specimens. However, implementing PCR-based diagnostics may be challenging in low-resource settings due to the relatively high costs of molecular equipment, reagents, maintenance, and technical expertise. Therefore, although molecular methods may offer greater diagnostic sensitivity, microscopy remains an important, more accessible diagnostic approach in resource-limited settings. A combination of conventional microscopy for routine screening and molecular methods for confirmation or epidemiological investigations may provide a more feasible diagnostic strategy in such settings.

Species-specific analyses indicated a higher prevalence of *S. nesbitti* (2.81%) compared with *S. hominis* (0.47%) and unidentified *Sarcocystis* spp., although species identification was not performed consistently across studies. Infection with *S. nesbitti* is believed to involve snakes as definitive hosts, with humans acting as accidental intermediate hosts [7,8,9]. Transmission to humans has been suggested to occur through the consumption of water contaminated with snake feces [14]. In contrast, humans serve as the definitive host for *S. hominis*, while cattle act as the intermediate host [3]. The lower prevalence of *S. hominis* and unidentified *Sarcocystis* spp. observed in this meta-analysis may be partly explained by the relatively low infection rates in humans acting as definitive hosts and by the reliance on fecal samples, which may underestimate infection [12,24]. Moreover, infections with *Sarcocystis* spp. and *S. hominis* are often asymptomatic, unlike *S. nesbitti* infections, which are more frequently associated with clinical manifestations [1]. This difference may partly explain the observed variation in pooled prevalence among *Sarcocystis* species.

In addition to human infections, *Sarcocystis* spp. have recently been reported in food products, including hamburgers [33,34], raw vegetables [35], sausages [36,37], and other meat products [38,39,40,41,42]. These reports suggested that the presence of the *Sarcocystis* spp. in raw or undercooked meat products indicates the potential risk associated with inadequate food processing and hygiene practices. Furthermore, *Sarcocystis* spp. have been detected in environmental samples such as soil contaminated with feces [43,44] and groundwater [45]. These findings suggest that contamination of soil and water sources indicates possible environmental persistence and fecal–oral transmission pathways.

This study has several limitations. First, although visual inspection of the funnel plot suggested asymmetry, Egger’s test did not indicate statistically significant small-study effects. However, publication bias cannot be definitively excluded because only 17 studies were included, and the power of tests for small-study effects is limited when the number of studies is small. This suggests that the observed asymmetry is more likely attributable to between-study heterogeneity rather than publication bias. Second, the substantial heterogeneity among studies was very high (*I*^2^ = 98.2%), which reduces the reliability and interpretability of the pooled prevalence estimate. This heterogeneity may reflect differences in study populations, geographic settings, study designs, diagnostic methods, and sampling strategies. Third, most studies were conducted in Asia, particularly in a limited number of countries, which may restrict the generalizability of the findings to other geographic regions and populations. Fourth, the limited number of eligible studies (17 studies) restricted the ability to perform reliable meta-regression analyses and to identify the factors responsible for the observed heterogeneity. Fifth, substantial variability in diagnostic approaches, including microscopy, PCR, and immunofluorescent antibody testing, may have contributed to differences in prevalence estimates. Sixth, excluding studies published before 2000 may have omitted earlier epidemiological evidence and influenced the estimated prevalence. Seventh, species identification was not consistently performed across studies, limiting species-specific comparisons and potentially contributing to the inclusion of unidentified *Sarcocystis* infections in the analysis. Eighth, the risk-of-bias assessment indicated that only three studies were considered to have a low risk of bias, while the remaining studies had some concerns or a higher risk of bias, which may affect the confidence in the pooled estimates. Ninth, the predominance of cross-sectional studies limits causal interpretation and prevents assessment of temporal relationships between potential risk factors and infection. Finally, many studies relied on microscopy-based detection, which may have underestimated the true prevalence because of its lower sensitivity compared with molecular methods.

## 5. Conclusions

This systematic review and meta-analysis suggest that human *Sarcocystis* infection is relatively uncommon but vary geographically. However, the limited number of included studies and their uneven geographical distribution, with most studies conducted in Asia, restrict the generalizability of the pooled prevalence estimates. The higher prevalence reported in some Asian countries may be influenced by the regional distribution of *S. nesbitti* and its definitive hosts, as well as differences in study populations, diagnostic methods, and sampling strategies. Further well-designed, population-based studies using standardized and sensitive diagnostic methods, particularly in underrepresented regions, are needed to better determine the global burden, geographical distribution, and public health significance of human sarcocystosis.

## Data Availability

All data relating to the present study are available in this manuscript, as well as in Tables S1–S4.

## Supplementary Materials

The following supporting information can be downloaded at: https://www.mdpi.com/article/10.3390/tropicalmed11090241/s1, Table S1. Search terms. Table S2. Details of included studies. Table S3. Methodological quality of the included studies. Table S4. Meta-regression and subgroup analysis.
