# Supplementary material for: Global Prevalence and Distribution of Human Sarcocystosis: A Systematic Review and Meta-Analysis
Source: Trop Med Infect Dis. 2026 Aug 25;11(9):241. doi: 10.3390/tropicalmed11090241 (PMC13611245; doi:10.3390/tropicalmed11090241)
Supplement: Supplementary file 1 [file tropicalmed-11-00241-s001.zip › Table S1. Search terms.pdf]

Table S1. Search terms

General keywords

(Sarcocystis OR Sarcocysti OR Sarcosporidia OR Sarcosporidias) AND (human OR patients)

PubMed 30 September 2025

No.	Key concept	Search terms	Results
1.	Sarcocystis	Sarcocystis[MeSH Terms] OR Sarcocysti[MeSH Terms] OR Sarcosporidia[MeSH Terms] OR Sarcosporidias[MeSH Terms] OR Sarcocystis[Text Word] OR Sarcocysti[Text Word] OR Sarcosporidia[Text Word] OR Sarcosporidias[Text Word]	2,525
2.	Human	human[MeSH Terms] OR patients[MeSH Terms] OR human[Text Word] OR patients[Text Word]	24,954,357
3.	1 AND 2	#1 AND #2	424

Scopus 30 September 2025

No.	Key concept	Search terms	Results
1.	Sarcocystis	TITLE-ABS-KEY (Sarcocystis OR Sarcocysti OR Sarcosporidia OR Sarcosporidias)	3,310
2.	Human	TITLE-ABS-KEY (human OR patients)	30,789,454
3.	1 AND 2	(TITLE-ABS-KEY (Sarcocystis OR Sarcocysti OR Sarcosporidia OR Sarcosporidias)) AND (TITLE-ABS-KEY (human OR patients))	716

Ovid (Journal) 30 September 2025

No.	Key concept	Search terms	Results
1.	Sarcocystis AND Human	(Sarcocystis OR Sarcocysti OR Sarcosporidia OR Sarcosporidias) AND (human OR patients)	23

Nursing & Allied Health Premium 30 September 2025

No.	Key concept	Search terms	Results
1.	Sarcocystis AND Human	(Sarcocystis OR Sarcocysti OR Sarcosporidia OR Sarcosporidias) AND (human OR patients)	208

Web of Science 30 September 2025

No.	Key concept	Search terms	Results
1.	Sarcocystis AND Human	(Sarcocystis OR Sarcocysti OR Sarcosporidia OR Sarcosporidias) AND (human OR patients)	462

Google Scholar 30 September 2025

No.	Key concept	Search terms	Results
1.	Sarcocystis AND Human	Sarcocystis AND Human	18,500 (Screening for the first 200 results)

Google Scholar (n = 200)

175 non-related articles

25 related articles

8 were excluded with reasons;

- Reviews (n = 4)

- Book or book chapter (n = 1)

- No records (n = 2)

- Conference abstract (n = 1)

Included (n = 17)
