# Supplementary material for: Global Prevalence and Distribution of Human Sarcocystosis: A Systematic Review and Meta-Analysis
Source: Trop Med Infect Dis. 2026 Aug 25;11(9):241. doi: 10.3390/tropicalmed11090241 (PMC13611245; doi:10.3390/tropicalmed11090241)
Supplement: Supplementary file 1 [file tropicalmed-11-00241-s001.zip › Table S4. Meta-regression and subgroup analysis.pdf]

Table S4. Meta-regression and subgroup analysis

1. Meta-regression analysis of the pooled prevalence of *Sarcocystis* infections

**Covariates**	**tau^2**	**H^2**	**Residual heterogeneity I^2 (%)**	**Test of moderators, *P* value**	**Number of datasets**
Continent	3.0668	21.24	95.29	0.4010	19
Country	0.7052	2.46	63.21	< 0.0001	19
Participants	2.6556	22.45	95.55	0.5996	19
Age group	3.0192	26.28	96.19	0.4781	19
Method for *Sarcocystis*	1.6866	16.77	94.04	0.0133	19
Sarcocystis species	2.5709	29.41	96.60	0.2354	19
Types of *Sarcocystis* infections	2.6701	22.99	95.65	0.0927	19

2. Subgroup analysis of the pooled prevalence of *Sarcocystis* infections

**Pooled prevalence**	**Subgroup**	**Test for subgroup difference**	**Pooled prevalence (%) [95% CI]**	**I^2 (%)**	**Number of datasets**
Continent		0.0067			
	Asia		0.94 [0.34 – 2.55]	86.8	15
	South America		7.14 [2.71 – 17.54]	N/A	1
	Europe		0.38 [0.02 – 5.52]	N/A	3
Country		< 0.0001			
	Malaysia		2.12 [0.59 – 7.35]	83.9	6
	Iran		0.17 [0.05 – 0.52]	0.0	4
	Thailand		0.96 [0.21 – 4.30]	63.4	4
	Cambodia		9.99 [8.34 – 11.93]	N/A	1
	Mexico		7.14 [2.71 – 17.54]	N/A	1
	France and Luxembourg		0.06 [0.01 – 0.45]	N/A	1
	Italy		10.0 [4.56 – 20.53]	N/A	1
	Belgium		0.10 [0.08 – 0.13]	N/A	1
Participants		< 0.0001			
	Symptomatic college students and teachers		3.31 [1.50 – 7.18]	0.0	2
	Immunodeficient individuals		0.13 [0.02 – 0.95]	N/A	1
	Villagers		1.46 [0.30 – 6.88]	87.6	5
	Patients in hospital/clinics		1.23 [0.24 – 6.18]	99.0	7
	Healthy people		0.00 [0.00 – 1.00]	N/A	1

	Schoolchildren		0.12 [0.02 – 0.83]	N/A	1
	Preschool-aged children		0.71 [0.18 – 2.79]	0.0	2
Age groups		0.0060			
	Children		0.71 [0.18 – 2.79]	0.0	2
	Adult		7.14 [2.71 – 17.5]	N/A	1
	All age ranges		0.53 [0.13 – 2.21]	99.2	8
	Not specified		1.24 [0.28 – 5.22]	90.0	8
Detection method for *Sarcocystis*		< 0.0001			
	PCR		3.31 [1.50 – 7.18]	0.0	2
	Microscopic method/PCR		0.09 [0.02 – 0.35]	0.0	2
	Microscopic method		0.61 [0.19 – 1.97]	98.7	12
	Histology/PCR		1.47 [0.21 – 9.71]	N/A	1
	Semi-nested PCR/sequencing		10.0 [4.56 –20.5]	N/A	1
	Immunofluorescent antibody test		16.0 [11.5 – 21.8]	N/A	1
Sarcocystis species		< 0.0001			
	S. nesbitti		2.81 [1.35 – 5.78]	0.0	3
	S. cruzi		0.13 [0.02 – 0.95]	N/A	1
	S. hominis		0.47 [0.06 – 3.35]	86.3	5
	Sarcocystis spp.		1.62 [0.39 – 6.41]	99.1	8
	Not assessed (0 infected-case)		0.11 [0.02 – 0.80]	N/A	2
Types of *Sarcocystis* infections		< 0.0001			
	Intestinal		0.0054 [0.0018; 0.0165]	98.4	15
	Muscular		0.0281 [0.0135; 0.0578]	0.0	3
	Not specified (blood samples)		0.1600 [0.1154; 0.2175]	N/A	1

N/A, not assessed
